# Testosterone regulation of sex steroid-related mRNAs and dopamine-related mRNAs in adolescent male rat substantia nigra

**DOI:** 10.1186/1471-2202-13-95

**Published:** 2012-08-06

**Authors:** Tertia D Purves-Tyson, David J Handelsman, Kay L Double, Samantha J Owens, Sonia Bustamante, Cynthia Shannon Weickert

**Affiliations:** 1 Schizophrenia Research Institute, Sydney 2021, Australia; 2 Neuroscience Research Australia, Sydney 2031, Australia; 3 School of Medical Sciences, University of New South Wales, Sydney 2031, Australia; 4 ANZAC Research Institute, Concord, 2139, Australia; 5 Bioanalytical Mass Spectroscopy Facility, University of New South Wales, Sydney 2031, Australia; 6 School of Psychiatry, University of New South Wales, Sydney 2031, Australia

**Keywords:** Sex steroids, Midbrain, Estrogen receptor, Androgen receptor, Gene expression, Adolescence, Schizophrenia

## Abstract

**Background:**

Increased risk of schizophrenia in adolescent males indicates that a link between the development of dopamine-related psychopathology and testosterone-driven brain changes may exist. However, contradictions as to whether testosterone increases or decreases dopamine neurotransmission are found and most studies address this in adult animals. Testosterone-dependent actions in neurons are direct via activation of androgen receptors (AR) or indirect by conversion to 17β-estradiol and activation of estrogen receptors (ER). How midbrain dopamine neurons respond to sex steroids depends on the presence of sex steroid receptor(s) and the level of steroid conversion enzymes (aromatase and 5α-reductase). We investigated whether gonadectomy and sex steroid replacement could influence dopamine levels by changing tyrosine hydroxylase (TH) protein and mRNA and/or dopamine breakdown enzyme mRNA levels [catechol-*O*-methyl transferase (COMT) and monoamine oxygenase (MAO) A and B] in the adolescent male rat substantia nigra. We hypothesized that adolescent testosterone would regulate sex steroid signaling through regulation of ER and AR mRNAs and through modulation of aromatase and 5α-reductase mRNA levels.

**Results:**

We find ERα and AR in midbrain dopamine neurons in adolescent male rats, indicating that dopamine neurons are poised to respond to circulating sex steroids. We report that androgens (T and DHT) increase TH protein and increase COMT, MAOA and MAOB mRNAs in the adolescent male rat substantia nigra. We report that all three sex steroids increase AR mRNA. Differential action on ER pathways, with ERα mRNA down-regulation and ERβ mRNA up-regulation by testosterone was found. 5α reductase-1 mRNA was increased by AR activation, and aromatase mRNA was decreased by gonadectomy.

**Conclusions:**

We conclude that increased testosterone at adolescence can shift the balance of sex steroid signaling to favor androgenic responses through promoting conversion of T to DHT and increasing AR mRNA. Further, testosterone may increase local dopamine synthesis and metabolism, thereby changing dopamine regulation within the substantia nigra. We show that testosterone action through both AR and ERs modulates synthesis of sex steroid receptor by altering AR and ER mRNA levels in normal adolescent male substantia nigra. Increased sex steroids in the brain at adolescence may alter substantia nigra dopamine pathways, increasing vulnerability for the development of psychopathology.

## Background

Androgens and estrogens produced by the gonads influence brain function including cognitive, motor and motivational behaviors and neurotransmitter release in midbrain dopamine circuits. Schizophrenia, characterized by hallucinations and delusions resulting from hyperactivity of the subcortical dopaminergic system (especially dorsal caudate) [1,2], affects males more severely than females [3] with the highest rate of onset seen in adolescent males [4]. Male adolescence involves a rapid increase in testosterone production leading to increased blood testosterone levels [5]. The increased risk of schizophrenia in male adolescents suggests that testosterone may directly induce transcriptional and maturational changes in midbrain dopamine neurons. However, molecular evidence supporting that sex steroids can change gene expression during the adolescent maturation of midbrain dopamine neurons does not currently exist.

Sex steroids activate ligand-dependent nuclear receptors. Testosterone initiates changes in gene expression via direct activation of androgen receptors (AR) or can interact with estrogen receptors after aromatization. Testosterone can be converted to the non-aromatisable androgen, dihydrotestosterone (DHT) by 5α-reductase enzymes (5αR) [6]. DHT is a pure androgen with greater potency at AR than testosterone and can initiate gene expression only via AR. Testosterone is also converted to estradiol by the cytochrome p450 enzyme, aromatase (*Cyp19A1*), to activate nuclear estrogen receptors, ERα and ERβ. All three sex steroid receptors (ERα, ERβ and AR) exist in adult rodent midbrain [7-11]. Two 5αR isozymes, 5αR-1 and 5αR-2, are expressed in rodent and human brain during adulthood [12] and aromatase is expressed in the midbrain [13]. Androgens differentially control 5αR mRNA levels in the rat prefrontal cortex [14] and high levels of 5αR activity have been detected in rat midbrain homogenates [15]. However, it is unknown whether testosterone can regulate the enzymes involved in steroid conversion in the adolescent male substantia nigra. We hypothesized that testosterone may change aromatase and 5αR mRNAs to alter sex steroid signaling over the period of adolescence in male rat substantia nigra.

Studies in rodents disagree as to how sex steroids modulate midbrain dopamine circuits. Some studies in adult male rats suggest that dopamine neurons are inhibited by circulating testosterone, as castration caused increased striatal dopamine release, increased locomotion and augmented the behavioral response to dopamine-potentiating drugs of abuse [16-18]. Other studies suggest the opposite, where exogenous testosterone can increase striatal dopamine levels and dopamine turnover in adult male rats [19,20]. Anatomically, testosterone can increase dopaminergic neuron axon density in targets [7], but may also lead to a decreased number of dopamine neurons in the midbrain [21]. Most studies have been carried out in adult rodents, but studies focusing on the adolescent period of brain development are imperative to understand the involvement of sex steroids in psychopathologies that emerge (or worsen) during adolescence.

In our study, we investigated whether gonadectomy and exogenous androgens or estradiol are able to influence dopamine neurons by changing tyrosine hydroxylase (TH) levels, the rate limiting enzyme in the production of dopamine and/or by changing the levels of mRNA encoding for enzymes involved in dopamine metabolism such as catechol-*O*-methyl transferase (COMT) and monoamine oxygenase (MAO) A and B in the substantia nigra (SN) of adolescent male rats. We also tested the extent to which sex steroid receptor mRNA expression changes in response to testosterone and estradiol in male adolescence in the substantia nigra.

## Methods

### Animal experiments

All animal experiments were approved by the Animal Care and Ethics Committee of the University of New South Wales in accordance with the National Health and Medical Research Council of Australia’s Code of Practice for the Care and Use of Animals for Experimental Purposes, which also conforms to standard international guidelines. Male Sprague–Dawley rats were used for all experiments (Animal Resource Centre, Perth, WA, Australia). Rats were group housed (3-4/cage) in 12/12 hr light/dark phases with constant humidity and temperature and free access to water and standard rat chow.

### Gonadectomy and sex steroid replacement

Adolescent male rats were gonadectomised at 45 days of age, prior to the adolescent testosterone increase, and given continuous replacement testosterone (T), dihydrotestosterone (DHT) or 17β-estradiol (estradiol, E) by subdermal silastic implant [22-24] for two weeks. By this age, intact animals have experienced the increase in testosterone associated with adolescence. There were five groups (~15 rats per group): intact (Intact), gonadectomy alone (Gdx); gonadectomy plus testosterone (Gdx+T); gonadectomy plus DHT (Gdx+DHT), gonadectomy plus estradiol (Gdx+E). Male rats (45 days old) were anaesthetized with an intraperitoneal (i.p.) injection of ketamine hydrochloride (60 mg/kg) and xylazine hydrochloride (10 mg/kg) (Provet, Castle Hill, Australia). The intact animals underwent sham abdominal surgery but gonads were left in place. Silastic implants were placed under the skin between the shoulder blades at time of gonadectomy. Gdx and Intact groups were given empty implants. Implants were 1 cm long, internal diameter 1.47 mm, outer diameter 1.95 mm (ends sealed with silastic adhesive). These implants have been characterized in previous studies and achieve supraphysiological, steady-state hormone levels and maintain seminal vesicle weights equal to that in untreated animals [23,25]. Seminal vesicles depend on androgen action for normal development and maintenance of structural and functional integrity [26]. Weight of seminal vesicles at the end of experiment served as an index of the restoration of androgen action by the implants as previously reported [25].

At 60 days of age rats were anaesthetized with 60 mg/kg sodium pentobarbital (Euthal, Delvet, Seven Hills, Australia). Brain was removed from the skull and a tissue block containing the midbrain was dissected following the Rat Brain Atlas as a guide [27]. The block was trimmed transversely at the cerebral aqueduct and two lateral segments of midbrain on either side of the ventral tegmental area (VTA) containing the SN were collected. Trunk blood was collected on day of euthanasia in 0.8 ml serum gel tubes (Z serum sep MiniCollect tube, Greiner Bio One, Wemmel, Belgium) and serum collected by centrifugation. Left and right hemisphere midbrain segments including SN were randomly assigned for either protein or RNA extraction.

### Immunohistochemistry

For immunohistochemical studies five normal intact male rats (50–53 days old) were deeply anaesthetized with pentobarbital (Euthal) followed by transcardial perfusion with 4% paraformaldehyde (PFA, pH 7.2). Midbrain was dissected in the coronal plane and post-fixed (4% PFA) overnight, washed with phosphate buffered saline (PBS) and cryoprotected in 30% sucrose/PBS.

Midbrain sections (40 μm) were collected in PBS and sections from each brain between Bregma −5.52 and −5.64 mm was incubated for 1 h with 10% horse serum (Sigma) and 0.1% Triton X-100 (Sigma) in PBS. This was followed by incubation with primary antibodies overnight at 4°C, washes and incubation with fluorescence-conjugated secondary antibodies for 2 h at room temperature. The antisera used were: TH (host species mouse, 1:500; MAB318, Millipore, Temecula, CA, USA) or TH (host species rabbit, 1:500; AB152, Millipore), ERα (host species mouse, 1:50; 6 F-11, Nova Castra, Leica-Microsystems, Wetzlar, Germany) and AR (host species rabbit, 1:200, PA1-110, Thermo Scientific, Australia, Scoresby, Victoria). ERβ immunohistochemistry was not performed as there are currently no well-validated commercial ERβ antibodies available. Primary antibody binding was visualized with fluorophore-conjugated species-specific secondary antibodies (donkey-anti-mouse-Alexa Fluor488 and 594, donkey-anti-rabbit-Alexa Fluor488 and 594, all 1:500, Life Technologies, Carlsbad, CA, USA). Sections incubated with only secondary antibody or with each primary antibody with the alternate (incorrect) secondary antibody confirmed that no fluorescent signal was due to non-specific or cross-over binding. Nuclei were visualized by incubation with 1 μg/ml DAPI (Sigma). Sections were mounted with glycerol/PBS (Citifluor AF1, London, UK). Sections were viewed under a Nikon Eclipse 80i fluorescence microscope (Nikon) with a CX9000 digital camera attached (MBF Biosciences, Williston, VT, USA). SN and VTA were demarcated under low power (4x) using StereoInvestigator (v8.27, MBF Biosciences) and referring to a Rat Brain Atlas [27]. Under higher power (20x) using StereoInvestigator, TH positive cells (100–200) were identified within the SN pars compacta, they were then assessed for receptor immunoreactivity and percentage of TH positive neurons expressing either receptor was determined. Images were also collected on a Nikon C1 laser scanning confocal unit (Nikon D-Eclipse C1, Nikon Australia) attached to a fluorescence microscope (Nikon Eclipse 90i). Images were acquired digitally and processed using the software EZ-C1 (v 3.5) for Nikon C1 confocal microscopes (Nikon Australia).

### Immunoblotting

The substantia nigra blocks were homogenized [0.1 M Tris, pH 7.5, 50% glycerol, proteinase inhibitor cocktail (Sigma Cat# P8340) and aprotinin (0.015 mM, Sigma, St Louis, MO, USA)] using a handheld electric homogenizer (Polytron, Kinematica, Lucerne, Switzerland). Protein concentration was determined using the Bradford protein assay (Sigma). An aliquot of each sample was combined and used as a standard and run in duplicate on each gel to allow standardization between blots (internal control). Standard curves with between 0.5 and 20 μg SN protein were run and TH expression was determined to be within a linear range using 3 μg protein. SDS-PAGE (10% acrylamide) was performed on 3 μg of protein per sample. Proteins were transferred to nitrocellulose (45 μm, Biorad, CA, USA). Primary antibodies were anti-TH (host species mouse, 1:5000; Chemicon) and anti-β-actin (host species rabbit, 1:5000; MAB1501, Millipore). Secondary antibodies were goat anti-mouse or anti-rabbit horseradish peroxidase (HRP) conjugated (1:4000, Millipore). Immunoreactive bands were detected using the LumiGlo detection kit (LumiGlo Reagent; Cell Signaling, Danvers, MA, USA) on hyperfilm (Amersham, GE Healthcare, Uppsala, Sweden). The immunoblots were scanned and band densities converted to numerical values using ImageJ software (ImageJ 1.43u, National Institutes of Health, USA). Relative intensity of TH bands were normalized to relative intensity of β-actin bands.

### RNA Extraction and cDNA Synthesis

Total RNA was extracted from substantia nigra samples in 800–1000 μl TRIzol reagent (Life Technologies Inc., Grand Island, NY, USA) with a handheld electric homogenizor (Polytron) as recommended by the manufacturer. RNA was quantified using a ND-1000 Spectrophotometer (Nanodrop Technologies, Wilmington, USA). RNA integrity (RIN) was assessed with high resolution capillary electrophoresis (Agilent Bioanalyzer 2100, Agilent Technologies, Palo Alto, CA, USA). Two separate aliquots of 3 μg RNA from each sample were reverse transcribed to cDNA with SuperScript III First-Strand Synthesis Supermix and random hexamers, according to the manufacturer’s protocol (Life Sciences). A parallel reaction was performed without reverse transcriptase as a negative control. The 2 aliquots of cDNA from each sample were pooled and diluted for RT-PCR.

### Quantitative Real-Time PCR (qPCR)

Messenger RNA (mRNA) levels of genes of interest were measured by TaqMan Gene Expression Assays (Applied Biosystems, Foster City, CA, USA) using an ABI Prism 7900HT Fast Real-Time PCR System and a 384-well format. Expression of three housekeeping genes, beta-glucuronidase (GusB), 18 S ribosomal RNA (18 S rRNA) and glyceraldehyde 3-phosphate dehydrogenase (GAPDH), was used to calculate the normalizing control for gene expression (termed geometric mean), and were selected on the basis that they were unchanged by the treatment. The Taqman probes used were: GusB (Rn00566655); 18 s rRNA (Hs99999901); GAPDH (Rn01775763). The geometric mean of the three housekeeping genes was calculated as described previously [28]. There were no group differences when the geomean of the three housekeepers was compared (F=1.67, df=4, *p*=0.168). Genes of interest were targeted by the following Taqman probes (Applied Biosystems): TH (Rn00562500), COMT (Rn00561037), MAOA (Rn01430950), MAOB (Rn00566203), ERα (Rn01640372), ERβ (Rn00562610), AR (Rn00560747) and Cyp19A1 (Rn00567222), 5αR-1 (Rn00567064) and 5αR-2 (Rn00575595, 5αR-2 mRNA levels in midbrain were too low to be accurately quantitated). Samples were run alongside a seven point standard curve using serial dilutions of cDNA derived from midbrain RNA pooled from a subset of 25 rats (taken from all treatment groups). No template controls were included which produced no signal for any mRNA examined. Measurements were performed in triplicate. PCR cycling conditions were: 50°C for 2 minutes, 95°C for 10 minutes, 50 cycles of 95°C for 15 seconds and 60°C for 1 minute. PCR data were captured with Sequence Detector Software (SDS version 2.4, Applied Biosystems). SDS software plotted real-time fluorescence intensity and the threshold was set within the linear phase of the amplification profiles.

### Sex steroid measurements

Testosterone (T), dihydrotestosterone (DHT) and 17β-estradiol (estradiol, E) were quantified in extracts of serum using a stable isotope dilution liquid chromatography-tandem mass spectroscopy (LC-MS/MS) method as described [29] and adapted for rodents [30]. The limit of quantitation for this method is 0.025 ng/ml T, 0.1 ng/ml DHT and 5 pg/ml E. T and DHT were also measured in serum extracts using a gas chromatography MS (GC-MS) method adapted from Horning et al., [31] (data presented in additional material).

### Statistics

Unless otherwise stated statistical analyses were conducted using SPSS software (IBM SPSS Statistics, version 19) and *p* < 0.05 was considered statistically significant. Seminal vesicle and body weight (g) data are shown as mean ± standard error of the mean (SEM). Immunoblotting data are presented as percent change of relative intensity compared to the Gdx group ± SEM. Immunoblotting data were normalized to β-actin expression and population outliers removed by performing Grubbs test for outliers (GraphPad Software). Data was then analyzed by one-way ANOVA followed by Fisher’s least significant differences (LSD), *post hoc.*

QPCR data are presented as percent change of mRNA levels relative to the Gdx group ± SEM. Outlier detection of the triplicates obtained from the qPCR raw data was used to exclude measurement errors [32]. qPCR raw data was normalized by the geomean of the housekeepers. Population outliers were removed by performing Grubb’s test on the normalized data and data were analyzed by one-way ANOVA followed by Fisher’s LSD.

## Results

### Confirmation of sex steroid replacement by organ weight

**Figure 1:**
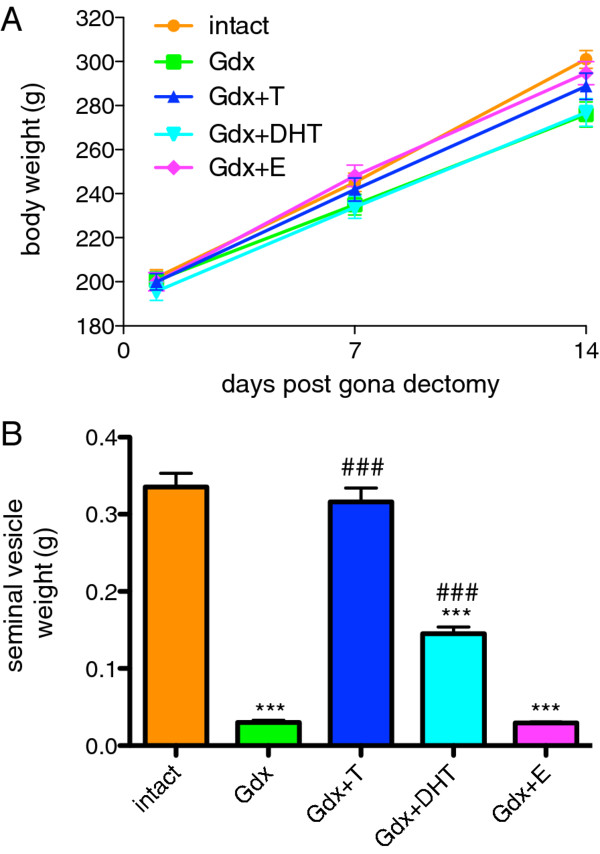
**Effect of gonadectomy and sex steroid replacement on body weights and seminal vesicle weights in adolescent male rats.(A)** There were no significant differences in overall growth between any of the five experimental groups (F=2.09, df=4, 221, *p* > 0.05). **(B)** Seminal vesicles weighed less following gonadectomy (n=16) compared with Intact rats (n=15) and this effect was completely prevented by T replacement (n=17), partly prevented by DHT (n=14) but unchanged by E (n=15). * denote comparisons with Intact, # denote comparisons with Gdx, ### and ***=p < 0.0001.

There were no significant differences in overall body growth between any of the five experimental groups (the mean - of all 5 groups - increase in body weight over the course of study was 44.0 ± 2.0%) (Figure 1A). There were no significant group differences in brain weight (results not shown). There was a significant effect of treatment group when examining seminal vesicle weights (F=149.2, df=4, *p* < 0.0001). Gdx reduced seminal vesicles to 9.0% weight of Intact group (Figure 1B). Seminal vesicles were maintained at 94.2% of intact weights by replacement testosterone and at 44% of intact weights by replacement DHT. Estradiol replacement had no effect on seminal vesicle weights such that the Gdx+E group was not significantly different to the Gdx group.

### Confirmation of sex steroid replacement by blood levels

DHT and T were measured by LC-MS/MS and GC-MS methods. Estradiol was detectable only by LC-MS/MS. Comparisons of GC-MS and LC-MS/MS androgen measurements in sera samples are included in additional material (see Additional Material File 1). As LC-MS/MS proved to require less serum (200 μl) and was able to detect circulating estradiol we report these measurements. Average circulating serum testosterone was 0.03 ± 0.001 ng/ml in the Gdx group (n=9), 2.8 ± 0.6 ng/ml (n=12) in the Intact group and 23.1±12.0 ng/ml in the Gdx+T group (n=14). Average circulating serum DHT was not detectable in the Gdx group (n=8), 0.2 ± 0.03 ng/ml in the Intact group (n=7), and 21.42 ± 10.6 ng/ml in the Gdx+DHT group (n=12). Circulating serum estradiol was 7.3 ± 0.15 pg/ml (duplicate measure of 8 pooled samples) in the Intact group and 17.0 ± 6.7 pg/ml in the Gdx+E group (n=8).

### Anatomical co-localization of AR and ER and TH in substantia nigra dopamine neurons

**Figure 2:**
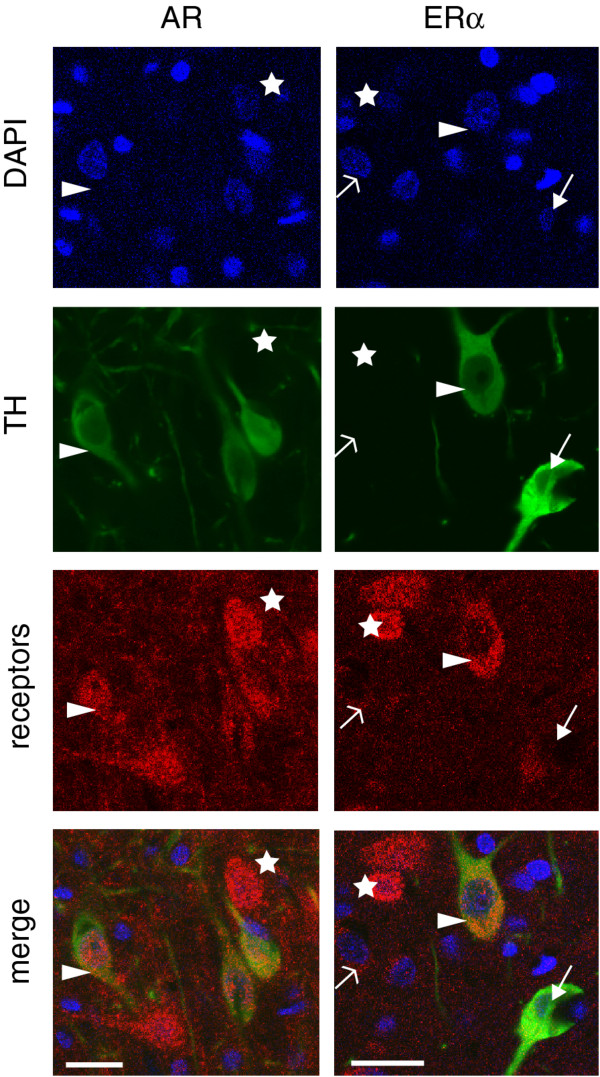
**AR and ERα immunoreactivity in dopamine neurons of the adolescent male rat substantia nigra.** Midbrain tissue sections (approximately bregma −5.52) were probed with antibodies raised against tyrosine hydroxylase (TH) and either androgen receptor (left column) or estrogen receptor α (right column). Blue staining indicates DAPI labeled nuclei (top row). Arrowheads indicate TH positive neurons (green) that are also immuno-positive for sex steroid receptors (red). Neurons with co-localized sex steroid receptors and TH immunostaining appear yellow in the merged images (bottom row, arrowheads). Stars indicate TH negative, sex steroid receptor positive (red) neurons, solid arrow indicates a TH positive, ERα negative neuron. Scale bar=20 μm.

Co-localization of TH and sex steroid receptors was indicated by AR immunoreactivity in ~65.5 ± 3.5% of TH positive (TH+) neurons and ERα-immunoreactivity in ~39.9 ± 4.4% of TH+ neurons in the normal intact male adolescent substantia nigra (50–53 days old, n=5) (Figure 2), indicating that many dopamine neurons in the substantia nigra could respond directly to sex steroids. Sex steroid receptor immunoreactivity was also present in TH negative cells.

### Regulation of substantia nigra TH protein by sex steroids

**Figure 3:**
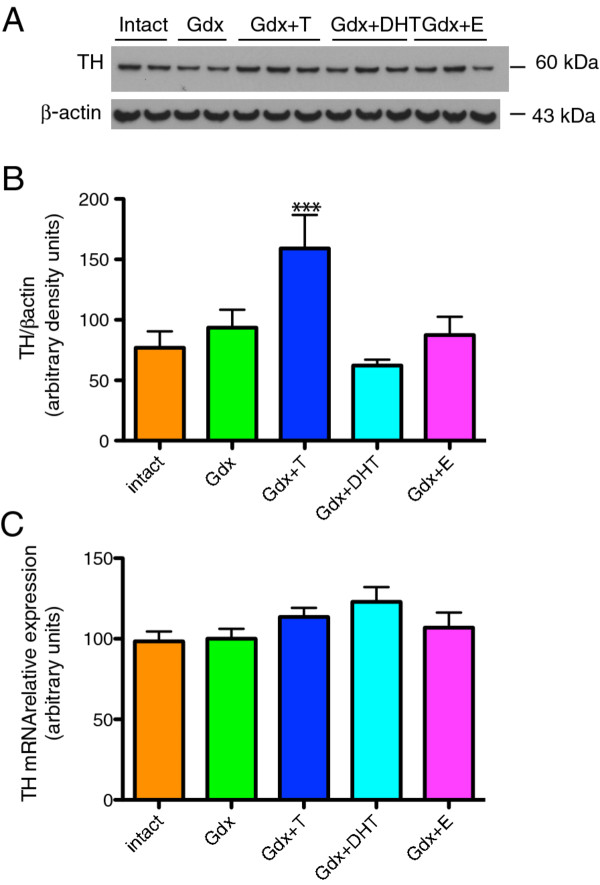
**Effect of gonadectomy and sex steroid replacement on TH protein and mRNA in adolescent male rats.(A)** Representative immunoblots of a subset of substantia nigra samples from all five groups showing TH protein (60 kDa) and β-actin protein (43 kDa). **(B)** TH protein was increased by testosterone (n=10) compared to Intact (n=10) and Gdx (n=10) groups, whereas DHT (n=8) and E (n=10) did not increase TH protein. Gdx+T was significantly different to Gdx+DHT and Gdx+E, ***=*p* < 0.001, and significantly different to Gdx and Intact, *p* < 0.05 and *p* < 0.01, respectively. **(C)** TH mRNA was measured using real time qPCR. Midbrain TH mRNA relative expression was not significantly different according to treatment group.

TH protein was detected at the expected molecular weight of 60 kDa and β-actin at 43 kDa (Figure 3A). There was a significant effect of treatment group on TH/β-actin protein levels in the substantia nigra in adolescent male rats (F=4.43, df=4, *p*=0.0043). TH protein in the substantia nigra was increased 70% in the Gdx+T group relative to the Gdx group and 106% relative to the Intact group (Figure 3B). There were no significant differences in TH protein levels between Intact, Gdx, Gdx+DHT or Gdx+E groups.

### Regulation of substantia nigra TH mRNA by sex steroids

There was no overall effect of treatment group on TH mRNA (F=1.97, df=4, *p*=0.109) in the SN of adolescent male rats. Fisher’s LSD *post hocs* were run as we hypothesized, *a priori,* that there would be an increase in TH mRNA with testosterone. There was a trend for TH mRNA to be increased in the Gdx+T group when compared to the Intact (13.6%) and Gdx (15.4%) groups (intact vs. Gdx+T; *p*=0.073; Gdx vs. Gdx+DHT, *p*=0.092) (Figure 3C).

### Modulation of dopamine metabolite enzyme mRNAs by sex steroids

**Figure 4:**
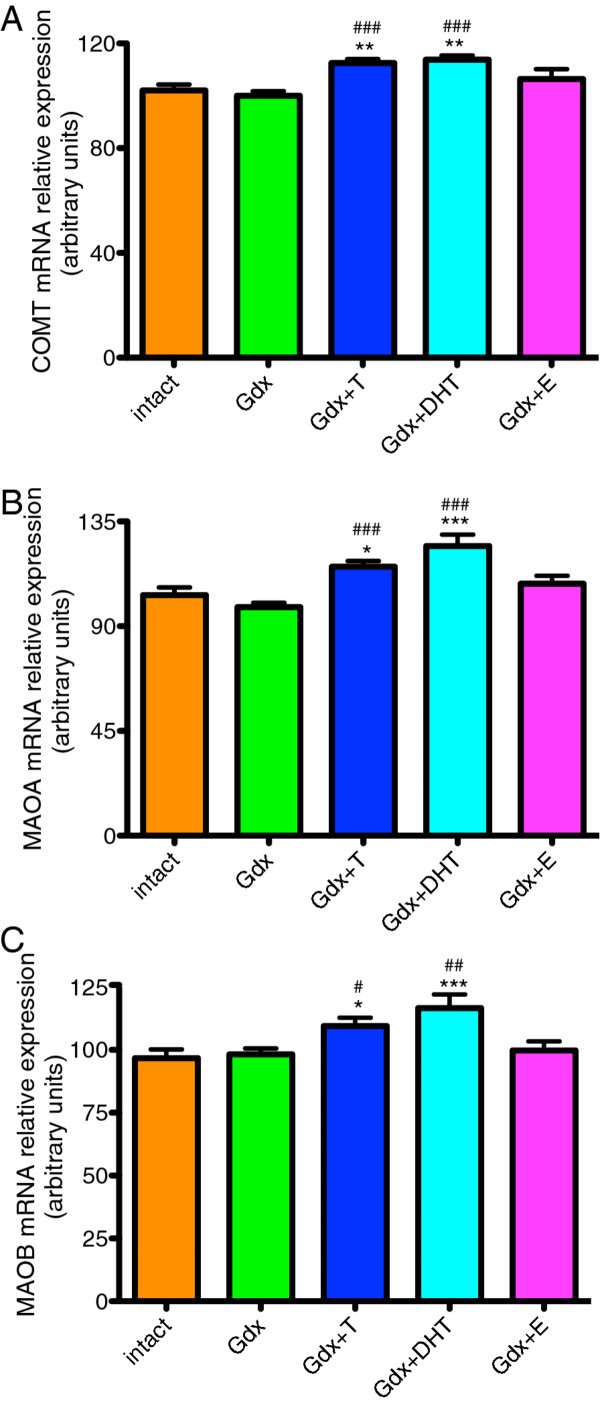
**Effect of gonadectomy and sex steroid replacement on dopamine metabolic enzyme COMT, MAOA and MAOB mRNA in the substantia nigra of adolescent male rats.(A)** COMT mRNA levels were significantly increased by T and DHT when compared to both Gdx and Intact groups. **(B)** MAOA mRNA and **(C)** MAOB mRNA levels were significantly increased by T and DHT when compared to both Gdx and Intact groups. * denotes comparisons with the Intact group, # denotes comparisons with the Gdx group. * and #=*p* < 0.05, ** and ##=*p* < 0.01, *** and ###=*p* < 0.0001.

There was a significant effect of treatment group on COMT (F=6.65, df=4, *p* < 0.0001), MAOA (F=9.983, df=4, *p* < 0.0001) and MAOB (F=5.35, df=4, *p* < 0.001) mRNA expression in the adolescent male rat substantia nigra. All three breakdown enzyme mRNAs were significantly increased in the Gdx+T and Gdx+DHT groups when compared to the Intact or Gdx groups (all p’s <0.05). None of the breakdown enzyme mRNA expression levels were changed in the Gdx+E group compared to the Intact or Gdx groups (Figure 4A, B, C).

### Modulation of androgen and estrogen receptor mRNAs by sex steroids

**Figure 5:**
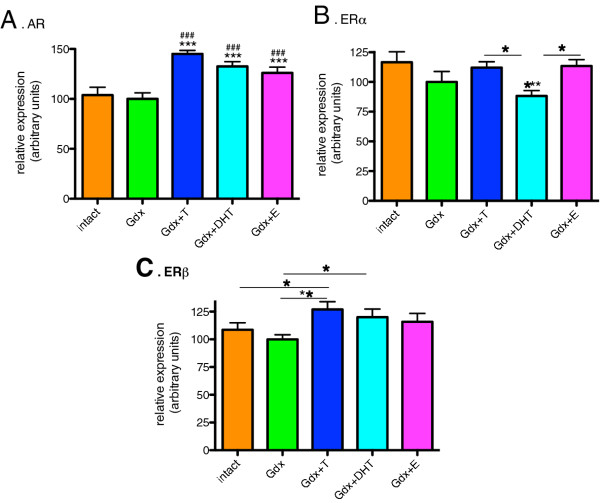
**The effect of gonadectomy and sex steroid replacement on sex steroid receptor mRNA expression in adolescent male rat substantia nigra.(A)** AR mRNA levels were increased by T, DHT and E compared to Intact (*) and Gdx (#) groups (n=12-14/group). **(B)** ERα mRNA was decreased by DHT replacement compared to the Intact group. T and E did not change ERα mRNA levels and were significantly different to the DHT treated group. **(C)** ERβ mRNA was increased by T (compared to Intact and Gdx groups) and by DHT (compared to Gdx only), but not by E. *=*p* < 0.05, **=*p* < 0.01, *** and ###=*p* < 0.0001.

We detected a significant effect of treatment group on AR mRNA expression (F=11.3, df=4, *p* < 0.0001) in the male SN. AR mRNA was increased by 45%, 32.5% and 26% in the Gdx+T, Gdx+DHT and Gdx+E groups, respectively, compared to the Gdx group and 39.8%, 27.7% and 21.4% in the Gdx+T, Gdx+DHT and Gdx+E groups, respectively, compared to the Intact group (Figure 5A). There was no significant difference in AR mRNA relative expression between the Intact and Gdx groups. We also found a significant effect of treatment on ERα mRNA expression (F=2.8, df=4, *p*=0.032) in the male SN. ERα mRNA was significantly decreased 24.3% in the Gdx+DHT group compared to the Intact group. There were no significant differences in ERα mRNA relative expression between GDX+T and GDX+E relative to the Gdx and Intact groups. The decrease in ERα mRNA relative expression (14.2%) in the Gdx group compared to the Intact group did not reach statistical significance (Intact vs. Gdx, *p*=0.089) (Figure 5B). We detected an effect of treatment group on ERβ mRNA expression in the male adolescent SN (F=2.6, df=4, *p*=0.045). ERβ mRNA expression was significantly increased in the Gdx+T (27%) and Gdx+DHT (20%) groups (Figure 5C).

### Modulation of sex steroid conversion enzyme mRNAs by sex steroids

**Figure 6:**
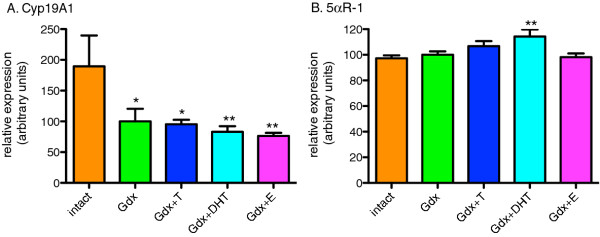
**The effect of gonadectomy and sex steroid replacement on steroid conversion enzyme mRNA expression in adolescent male rat substantia nigra.(A)**. Cyp19A1 mRNA was decreased by gonadectomy and this was not prevented by T, DHT or E replacement. * denotes comparisons with Intact, * = p < 0.05, ** = p < 0.01 **(B)**. 5αR-1 mRNA was increased by DHT compared to the Intact group but not by T or E replacement. ** p = < 0.01.

We found a significant effect of treatment group on Cyp19A1 mRNA (F=2.8, df=4, *p*=0.034) with decreased expression (40.4%) in the Gdx group compared to the Intact group and this was not reversed by replacement of any of the three sex steroids compared to Gdx and all replacement groups were significantly decreased compared to Intact group (Figure 6A). We also found a significant effect of treatment group on 5αR-1 mRNA expression (F=3.76, df=4, *p*=0.009). Relative expression of 5αR-1 mRNA was increased by 17.4% in the Gdx+DHT group when compared to the Intact group. There was a trend for 5αR-1 mRNA expression to be increased (9.7%) in the Gdx+T group compared to the Intact group (intact vs. Gdx+T, *p*=0.07) and 5αR-1 was unchanged by estradiol (Figure 6B).

## Discussion

Sex steroids have been implicated in the modulation of neurotransmitter systems, including dopamine, albeit with conflicting reports on the direction of change [16-20]. The psychotic symptoms associated with schizophrenia are thought to be driven by increased dopamine neurotransmission in subcortical brain regions [1,2], but the underlying causes of this hyperdopaminergia are unknown. The greater prevalence of schizophrenia in males, the peak of onset during adolescence, the increased symptom severity and the earlier age of onset relative to females [3,4,33,34] all point toward a contribution by testosterone, which increases during male adolescence, in the etiology of schizophrenia [35,36]. However, the mechanisms by which testosterone contributes to the disorder in males are uncertain. We report that TH protein as well as COMT, MAOA and MAOB mRNA expression levels in the substantia nigra of adolescent male rats are sensitive to changes in testosterone via androgenic mechanisms but appear to be relatively insensitive to changes in estrogen alone over the male adolescent period.

Our results demonstrate the presence of ERα and AR mRNA and protein and ERβ mRNA in the substantia nigra of adolescent rat midbrain. Our data are in agreement with previous studies demonstrating ERα [11,37] and AR [38] in midbrain dopamine neurons (although see [38]). Studies in adult male rats indicate that ERβ is also expressed in the substantia nigra [10,39] and many ERβ positive neurons are TH positive [40]. All three receptors have been identified in the substantia nigra in male rat pups up to postnatal day 30 (PN30) [11]. Our study highlights that dopamine neurons of the substantia nigra are able to respond directly to testosterone, or to testosterone converted to estradiol, during the male adolescent period.

Precisely how neurons respond to sex steroids will depend on the type, the combination and the levels of sex steroid receptor(s) and steroidogenic enzymes expressed and the nature of receptor activation via either 5α reduction and/or aromatization of testosterone. In our study, AR mRNA was positively regulated by all three sex steroids, implicating both androgenic and estrogenic mechanisms in the testosterone-induced up-regulation of midbrain AR gene expression. In contrast, estrogen receptor mRNAs were only changed by androgens and not by estrogen. We found opposite androgen regulation of ERα mRNA compared to ERβ mRNA where ERα mRNA was down-regulated by DHT and ERβ mRNA was up-regulated by testosterone and DHT. When co-expressed with ERα, via heterodimerization with ERα, ERβ can exert an inhibitory effect on ERα-mediated gene expression and in many instances opposes the actions of ERα [41,42]. Our results suggest that testosterone may co-ordinate simultaneous changes in sex-steroid receptors to increase responsiveness to testosterone through AR and ERβ and reduce responsiveness through ERα via decreased ERα and increased ERβ mRNAs.

Our data suggest that, during adolescence, testosterone may modulate dopaminergic tone within the substantia nigra by increasing levels of enzymes regulating both the production and metabolism of dopamine, suggestive of a local increase in dopamine utilization and turnover within this brain region. Our data suggest that this regulation of TH protein and COMT, MAOA and MAOB mRNAs is not mediated via ERs, but rather through AR. However, studies in ERα knock out mice showed reduced levels of TH in male and female midbrain, indicating that significant changes in TH can be mediated by loss of ERα [43]. In the knock out mouse, the ERα gene is changed from early development and the developmental trajectory driven by sex steroids of these neurons may be very different to animals with normal ERα expression. Studies in ERβ knockout mice show morphological abnormalities including neuronal shrinkage and neuronal loss in the substantia nigra indicating ERβ is important for midbrain neuronal survival [44]. Testosterone increased ERβ mRNA in our study, implying that testosterone can potentially contribute to ERβ mediated effects such as neuron survival and maturation.

We did observe that pure AR stimulation through DHT may lead to decreased TH protein levels relative to testosterone replacement suggesting testosterone action on TH protein levels may vary, depending on if only AR (DHT) or both AR and ERs (testosterone) are stimulated. However, the apparent lack of capacity for TH protein to be increased by DHT compared to T may also be due to a dose effect if an inverted U-shaped dose response curve of TH protein to AR stimulation exists, as DHT is more potent at the AR than T and equivalent replacement concentrations (~21 ng/ml) were achieved. This possibility would need to be tested with further studies.

Dendritic release of dopamine in the substantia nigra results in a feedback inhibition of dopamine neurons that regulates their excitability [45], increased levels of dopamine metabolic enzymes, COMT and MAOA and B, at the cell bodies in response to increased testosterone, as we report here, suggests a mechanism that may shorten the duration of feedback inhibition via released dopamine and thus regulate dopaminergic tone. In previous studies in adult male rats, although gonadectomy had no effect, testosterone replacement increased COMT activity in both cortex and striatum [46]. Testosterone attenuated the gonadectomy-induced increase in MAOA activity in the cortex, while MAOA activity was unchanged in the striatum and MAOB activity was unchanged in either region [46]. A study in intact adult male rats treated with injections of the androgen, nandrolone, reported decreased activity of MAOA and B with a low dose in the dorsal striatum whereas a high dose increased MAOB mRNA in the substantia nigra only and MAOA and COMT mRNA levels were unchanged in all regions [47]. Our data strengthen this existing data and demonstrate that testosterone can contribute to changes in regulatory potential of dopamine neurons in the substantia nigra. Our results highlight the potential for adolescence-driven increases in circulating testosterone to augment the capacity of midbrain neurons to synthesise and break down dopamine in the normal adolescent male rat substantia nigra. We propose that in individuals with an underlying susceptibility to schizophrenia, such as a genetic variation of the COMT gene [48,49], these androgen-driven increases in dopamine metabolic enzyme mRNAs may contribute to dopamine dysregulation in the substantia nigra at adolescence.

Other factors that may modify brain responses to increasing testosterone at adolescence include changing levels of steroidogenic enzymes. In adolescent male rats, we report increased levels of mRNA of the enzyme that converts T to DHT, 5αR-1, due to DHT (AR) but not estradiol (ERs). Increases in local levels of 5αR steroidogenic enzymes may increase local levels of non-aromatisable androgens (DHT) and serve to potentiate the increase in testosterone action via AR putatively creating a positive feedback loop. Gonadectomy resulted in decreased aromatase mRNA, responsible for converting T to E, in the midbrain, and this reduction was not reversed by replacement of any sex steroid. Thus, removing the male gonads appears to bias the midbrain toward more limited estradiol synthesis. Our data is in contrast to the hypothalamus, where testosterone is a major regulator of aromatase [50] suggesting significant regional differences exist. In the substantia nigra, decreased or unchanged levels of aromatase and increased levels of 5αR-1 in the midbrain could be expected to shift the balance of sex steroid signaling towards AR driven events, consistent with the AR-mediated induction of AR mRNA that we find. Inhibition of 5αR activity has been shown to counter several psychotic-like behavioral effects induced by apomorphine treatment in rats [51]. As testosterone increases with the onset of puberty more testosterone is available for reduction to DHT by 5αR-1, which in turn would be expected to increase the level of 5αR-1 mRNA. If, as suggested, increased 5αR (and therefore increased DHT) contributes to psychosis [52], this positive feedback loop may trigger psychosis in individuals with an underlying susceptibility towards hyperdopaminergia and schizophrenia.

Deficits in prepulse inhibition (PPI, a measure of sensorimotor gating that is regulated by dopamine) that result from an inability to appropriately integrate sensory information with a motor response, are associated with schizophrenia and degree of PPI deficit can be used as an index of schizophrenia-like behavior [53]. In contrast, we have found that PPI was attenuated during normal male adolescent development in primates [54]. Rhesus Macaques, that had undergone prepubertal gonadectomy had a robust PPI response, suggesting that the presence of adolescent testosterone may induce brain and behavioral changes which appear similar to what is found in schizophrenia (diminished PPI) and may increase manifestation of psychiatric-like symptoms. In rats, male castration did not change baseline PPI in adulthood, but serotonin receptor-stimulated changes in PPI differed in castrated rats compared to testosterone replaced rats [55]. Additionally, in adult male aromatase knockout mice (ArKO), which have higher levels of testosterone, baseline PPI is decreased with age when compared to controls [56]. Although PPI was not measured in our study, our results suggest that alterations in dopamine neurotransmission may contribute to the molecular basis for how changing testosterone may impact PPI during adolescence and confirm our observation of testosterone regulation of TH levels in the adolescent male monkey striatum [54].

In summary, treatment with exogenous sex steroids, be it T, DHT or E, shifted the adolescent male rat midbrain to a more androgen responsive state. In addition, increasing levels of circulating testosterone in male adolescence could be anticipated to increase AR mRNA, decrease ERα mRNA and increase ERβ mRNA. The underlying susceptibility to schizophrenia may involve the inability of the brain to respond appropriately to sex steroids [57], which is unmasked at adolescence. For example, either lower endogenous ERα [57] and/or increased AR signaling could be exaggerated by adolescent testosterone and thus, testosterone could shift the individual toward a dysregulated dopaminergic state. As yet, the levels of sex steroid receptors or sex steroid conversion enzymes have not been specifically measured in the midbrain of patients with schizophrenia.

## Conclusions

In conclusion, the mechanism of testosterone action in the adolescent male rat substantia nigra includes regulation of TH levels and dopamine metabolic enzymes as well as modulation of sex steroid signaling by controlling levels of sex steroid receptor and androgen activating enzyme gene expression. Understanding how testosterone action modulates synthesis of sex steroid receptors and androgen activation in the normal adolescent male substantia nigra increases our understanding of the potential role of sex steroids in dopamine neuron activity at adolescence and how sex steroids may contribute to psychopathology involving dopamine dysregulation.

## Abbreviations

18 S rRNA: 18S Ribosomal Ribonucleic Acid; 5αR: 5 alpha Reductase; ANOVA: Analysis Of Variance; AR: Androgen Receptor; ArKO: Aromatase Knockout; cDNA: Complementary Deoxyribonucleic Acid; COMT: Catechol-O-Methyl Transferase; DHT: Dihydrotestosterone; E: 17β-Estradiol; ER: Estrogen Receptor; GAPDH: Glyceraldehyde-3-Phosphate Dehydrogenase; Gdx: Gonadectomy; GusB: Beta-Glucuronidase; GUSB: Glucuronidase-β; HRP: Horse Radish Peroxidase; LC-MS/MS: Liquid Chromatography Tandem Mass Spectroscopy; MAO: Monoamine Oxidase; mRNA: Messenger Ribonucleic Acid; PBS: Phosphate Buffered Saline; PFA: Paraformaldehyde; PN: PostNatal; PPI: Prepulse Inhibition; qPCR: Quantitative real-time Polymerase Chain Reaction; RIN: Ribonucleic acid Integrity Number; RT: Room Temperature; RT-PCR: Reverse Transcriptase Polymerase Chain Reaction; SEM: Standard Error of the Mean; SN: Substantia Nigra; T: Testosterone; TH: Tyrosine Hydroxylase; VTA: Ventral Tegmental Area.

## Competing interests

The authors declare that they have no competing interests.

## Authors’ contributions

TPT helped conceive, design, coordinate and interpret the study and carried out all the experiments, except for the LC-MS/MS analysis of sera and dopamine breakdown enzyme qPCRs, and drafted the manuscript. DJH provided the silastic implants and the LC-MS/MS analysis and gave critical comments on the manuscript. SJO completed the dopamine breakdown enzyme qPCRs. SB provided GC-MS analysis of androgens in sera and provided comments on the manuscript. KLD helped conceive and design the study and helped draft the manuscript. CSW helped conceive, design, coordinate and interpret the study and edited the manuscript. All authors have read and approved the final manuscript.

## Supplementary Material

Additional file 1 **Comparison of LC-MS/MS and GC-MS measurements of circulating testosterone and DHT in rat sera.** Circulating androgens were measured in sera using two mass spectroscopy methods, GC-MS and LC-MS/MS. Correlations of testosterone and DHT levels measured by the two methods were performed. Testosterone levels measured by GC-MS and LC-MS/MS correlated (r=0.86, *p* < 0.0001). Click here for file
