# Supplementary material for: Testosterone regulation of sex steroid-related mRNAs and dopamine-related mRNAs in adolescent male rat substantia nigra
Source: BMC Neurosci. 2012 Aug 6;13:95. doi: 10.1186/1471-2202-13-95 (PMC3467168; doi:10.1186/1471-2202-13-95)
Supplement: Additional file 1 — Comparison of LC-MS/MS and GC-MS measurements of circulating testosterone and DHT in rat sera. Circulating androgens were measured in sera using two mass spectroscopy methods, GC-MS and LC-MS/MS. Correlations of testosterone and DHT levels measured by the two methods were performed. Testosterone levels measured by GC-MS and LC-MS/MS correlated (r=0.86, p < 0.0001). [file 1471-2202-13-95-S1.pdf]

A

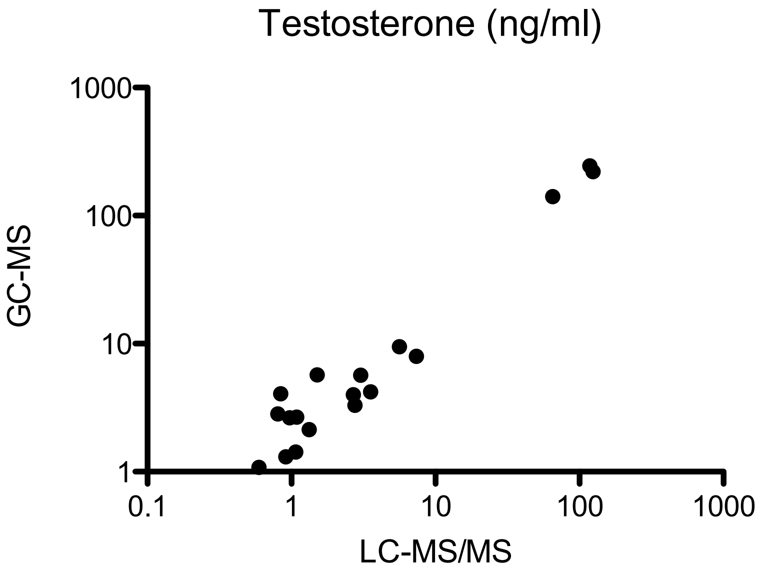

B

	Testosterone	DHT
	ng/ml (n)	ng/ml (n)
**Intact**	4.70 (10)	nd (10)
**Gdx**	1.00 (6)	nd (7)
**Gdx+T**	20.6 (8)	0.10 (8)
**Gdx+DHT**	1.70 (7)	68.0 (6)

Figure 1: Comparison of testosterone measured in sera by GC-MS or LC-MS/MS.
(A). Correlation of circulating testosterone in rat sera from Intact and Gdx+T groups ($P < 0.0001$, $r = 0.86$)
(B). Average circulating testosterone and DHT in sera quantified by GC-MS. nd = not detected.
